# DLGAP5 Drives Lung Adenocarcinoma Cell Growth by Promoting Autophagy Activity

**DOI:** 10.1155/carj/2731987

**Published:** 2026-06-08

**Authors:** Shuying You, Xiangbo Zeng, Na Li, Lile Wang

**Affiliations:** ^1^ Department of Respiratory Medicine, The Second People’s Hospital of Hunan Province/Brain Hospital of Hunan Province, Changsha, China; ^2^ Department of Respiratory Medicine, Hunan Provincial People’s Hospital/The First Affiliated Hospital of Hunan Normal University, Changsha, China, hunnu.edu.cn

**Keywords:** apoptosis, autophagy, DLGAP5, lung adenocarcinoma, proliferation

## Abstract

**Background:**

Autophagy is reported to participate in tumorigenesis and plays a vital role in tumor cell survival. Here, we aimed to elucidate the regulatory mechanism between discs large homolog‐associated protein 5 (DLGAP5) and autophagy in lung adenocarcinoma (LUAD) development.

**Methods:**

RT‐qPCR and western blot methods were utilized to assess DLGAP5 expression. Cell proliferation and apoptosis abilities were monitored utilizing Cell Counting Kit 8 (CCK‐8), EdU, and flow cytometry assays. Autophagy‐related proteins were quantified using western blot and immunofluorescence analysis. A xenograft tumor model was established to measure tumor growth.

**Results:**

DLGAP5 was significantly overexpressed in LUAD cell lines compared to 16HBE cells, especially in PC‐9 and A549 cells. Functional assays highlighted that loss of function of DLGAP5 repressed cell proliferation and triggered apoptosis both in vitro and in vivo, while DLGAP5 overexpression had the opposite effect. After DLGAP5 blockage, the autophagy‐related LC3II/I and Beclin1 expressions declined, while p62 expression increased. Moreover, the increased proliferation and autophagy activity and suppressed apoptosis mediated by DLGAP5 overexpression were greatly restrained after 3‐MA (an autophagy‐specific inhibitor).

**Conclusion:**

To sum up, DLGAP5 could contribute to proliferation and impede apoptosis of LUAD cells by activating autophagy activity, suggesting that targeting DLGAP5 might be a promising approach for LUAD treatment.

**Highlight**
1. Loss of function of DLGAP5 repressed proliferation and triggered apoptosis in LUAD cells.
2. DLGAP5 enhanced autophagy activity in LUAD cells.
3. The oncogenic role of DLGAP5 in LUAD was associated with increased autophagy activity.

## 1. Introduction

Lung cancer is an extremely heterogeneous malignancy with high morbidity and mortality globally, posing a significant threat to human health and quality of human life [1, 2]. It is estimated that there are approximately 1.76 million deaths and 2 million new cases of lung cancer each year [3]. Clinically, non–small‐cell lung cancer (NSCLC) accounts for approximately 85% of all lung cancer diagnoses, with lung adenocarcinoma (LUAD) being the most common major histological subtype [4]. In past decades, various therapeutic strategies, such as radiation, immunotherapy, and molecular targeted therapies, have become available for advanced LUAD patients’ treatment. However, the current 5‐year survival rate for patients is still less than 20% [5, 6]. The pathogenesis of LUAD is very complex and has not been fully elucidated so far. Thus, elucidating the specific molecular action pathway during LUAD initiation and development is vital for identifying helpful diagnostic and therapeutic targets.

Discs large homolog‐associated protein 5 (DLGAP5) is an essential member of the DLGAP family (DLGAP1∼5), also known as hepatoma cancer upregulated protein (HURP), disc large homolog 7 (DLG7), or KIAA0008. Physiologically, DLGAP5 is responsible for encoding a cell cycle–regulated microtubule‐associated protein, which is an effector of Ran GTP to involve in the regulation of mitotic centromere fibers [7]. Numerous studies have described that DLGAP5 plays a vital role in the initiation and development of malignant tumors [8–10]. For example, DLGAP5 has been reported as a mitosis‐associated gene in acute myeloid leukemia and positively correlated to poor prognosis and drug resistance [11] and was also involved in long‐term in vitro proliferation and cell cycle regulation [12]. Loss of function of DLGAP5 also greatly suppressed ovarian cancer cell proliferation and triggered G2/M phase arrest and apoptosis [13]. A recent study by Tang et al. uncovered that DLGAP5 exhibited a higher expression in LUAD patients, and the higher expression was positively associated with poor prognosis and immunotherapy [14, 15]. However, the detailed roles and action pathway of DLGAP5 in LUAD remain unclear.

Autophagy is a tightly regulated and evolutionarily conserved catabolic process that provides nutritional support and meets the energy demands for cells by decomposing unnecessary proteins, lipids, and organelles to maintain metabolic balance and homeostasis. Dysregulation of autophagy has been uncovered to participate in a series of diseases, including diabetes, neurodegenerative disease, cardiovascular disease, and malignant tumors [16, 17]. Specifically, autophagy exhibits a dual role in tumor biology, capable of both promoting and suppressing tumor in tumor cell survival. In most cases, autophagy supports the survival of tumor cells by resisting nutrient deprivation, hypoxia, and other adverse conditions [17]. In LUAD, autophagy activation has been reported to aggravate tumor growth, migration, invasion, immune infiltration, chemotherapy resistance, etc. [18–20], suggesting that autophagy inhibition is a promising strategy for LUAD treatment. However, the role of DLGAP5 in autophagy regulation remains rarely reported.

The present work was aimed to probe the underlying association between DLGAP5 and autophagy in LUAD development. The experimental findings revealed that DLGAP5 was highly expressed in LUAD cell lines and could activate autophagy activity, thus contributing to LUAD cell growth in vitro and in vivo. Finally, our work further elucidates the action pathway of DLGAP5 in LUAD pathogenesis and implies that DLGAP5 might be a promising therapeutic target in LUAD.

## 2. Methods

### 2.1. Cell Culture

Normal pulmonary epithelial cells (16HBE) were obtained from ATCC (Manassas, Virginia, USA), while the LUAD cell lines (CALU‐3, NCI‐H1975, A549, NCI‐H1299, and PC‐9) were supplied by the Chinese Academy of Sciences cell bank (Shanghai, China). All cell lines were cultivated at 37°C in a 5% CO_2_ incubator. PC‐9 cells were grown in DMEM medium (Gibco, Grand Island, USA). Beyond that, all cell lines were maintained in RPMI1640 medium. All culture medium was added with 10% FBS (Sigma‐Aldrich, Inc. St. Louis, MO, USA), 100 mg/mL streptomycin, and 100 U/mL penicillin.

### 2.2. Cell Transfection and Treatment

Short hairpin RNA of DLGAP5 (sh‐DLGAP5), DLGAP5 overexpressing vector (OV‐DLGAP5, pcDNA3.1 vector), and their negative controls (sh‐NC, OV‐NC) were provided by GenePharma (Shanghai, China). In short, A549 and PC‐9 cells (1 × 10^6^ cells/well) were placed into 6‐well plates and cultivated overnight. When cell reached confluence up to 80%–90%, the original medium was removed, the serum‐free medium was added, and then the above RNA molecules (50 nM) were transfected into cells by using Lipofectamine3000 (Thermo Fisher Scientific, Waltham, USA). To block autophagy activity, cells were treated with 3‐MA (10 mM, Sigma‐Aldrich) for 24 h.

### 2.3. Reverse Transcription–Quantitative Polymerase Chain Reaction (RT‐qPCR)

RNA samples were obtained from TRIzol reagent (Thermo Fisher Scientific). PrimeScript 1st strand cDNA synthesis kit (TaKaRa, Tokyo, Japan) was acquired to generate cDNA. SYBR Green reagent (TaKaRa) was performed to examine DLGAP5 expression by RT‐qPCR assay. The primer sequences were shown as follows (5′–3′): DLGAP5 (F) AAGTGGGTCGTTATAGACCTGA, (R) TGCTCGAACATCACTCTCGTTAT; GAPDH (F) CCTGTTCGACAGTCAGCCG, (R) GAGAACAGTGAGCGCCTAGT. The level of DLGAP5 was calculated by the 2^−ΔΔCt^ method and normalized to GAPDH.

### 2.4. Western Blot

After different treatments, cells were obtained and lysed in RIPA lysate buffer (Beyotime, Shanghai, China) to isolate total protein samples. After quantified using a BCA kit (Beyotime), equal amounts of protein were separated by 10% SDS‐PAGE and moved onto a PVDF membrane. Next, the membranes were blocked in 5% BSA for 60 min and incubated with primary antibodies including DLGAP5 (ab84509), LC3B (ab192890), Beclin1 (ab207612), p62 (ab109012), and β‐actin (ab8226) overnight at 4°C. On the next day, the membranes were incubated with the secondary antibody (ab28151) for another 60 min. ECL‐chemiluminescent kit (Amersham Pharmacia, UK) was employed to visualize immunoblots. Finally, immunoblots were imaged by a gel documentation system (Bio‐Rad, USA) and quantified by ImageJ software. All Western blot experiments were performed with at least three independent biological replicates. The primary antibodies in this investigation were all purchased from Abcam (Cambridge, MA, USA).

### 2.5. Proliferation Assay

Cell proliferation was examined by Cell Counting Kit 8 (CCK‐8) assay and EdU assays. The treated cells (5000 cells/well) were seeded into 96‐well plates and incubated for the indicated times. At each time point, 10 μL CCK‐8 (Sigma) was added to each well. After incubation for another 4 h, the absorbance of each well was recorded by using a microplate reader (Bio‐Tek). For the EdU assay, treated cells (5000 cells/well) were plated into a 96‐well plate and cultivated in an incubator at 37°C with 5% CO_2_. The following day, cells were further stained with EdU reagent (10 μM) for 2 h, fixed with 4% paraformaldehyde, and finally stained with Hoechst 33342. After washing, cells were captured and observed under a fluorescent microscope.

### 2.6. Apoptosis Assay

A commercial FITC Annexin V Apoptosis Detection Kit (KeyGen, Nanjing, China) was employed to examine the apoptotic rate. The treated cells were collected and washed with PBS for three times. Then, the mixture composed of 10 μL of Annexin V and 10 μL of PI was added into the cells for staining for 15 min. Finally, the cell apoptotic rate of cells was evaluated utilizing flow cytometry (FACScan; BD Biosciences).

### 2.7. Immunofluorescence

After the indicated treatment, PC‐9 and A549 cells were fixed with 4% paraformaldehyde for 30 min and then permeabilized with 0.5% Triton X‐100 for 20 min. Following blocking with 1% BSA, the primary LC3B antibody (Abcam, ab192890) was added to cells for incubation overnight at 4°C. The next day, the cells were further stained with the fluorophore‐conjugated secondary antibody at room temperature for 60 min. DAPI was applied to label cell nucleus. Finally, cells were imaged under a fluorescence microscope.

### 2.8. Xenograft Model

All experimental procedures were approved by the Second People’s Hospital of Hunan Province/Brain Hospital of Hunan Province. The BALB/c nude mice (5 weeks old) were provided by Hunan SJA Laboratory Animal Co., Ltd (Changsha, Hunan, China). All mice were randomly divided into two groups: the sh‐NC group and the sh‐DLGAP5 group (*n* = 5 per group). A549 cells (4 × 10^7^ cells/200 μL for each mouse) with transfected sh‐NC or sh‐DLGAP5 were subcutaneously injected into the right flank of each animal, respectively. Tumor volumes were determined every week until 5 weeks. Then, the mice were sacrificed to collect tumor tissues for next experiments.

### 2.9. Immunohistochemistry

Tumor tissues from each mouse were fixed in 4% formaldehyde overnight and washed and dehydrated in increased concentrations of ethanol solution. After clearing, tissues were embedded in paraffin and sectioned at 5‐μm‐thick slices. The slices were dewaxed and rehydrated and next incubated with 3% H_2_O_2_ to block endogenous peroxidase. Afterward, the slices were blocked by 10% (v/v) BSA, and the slice was incubated with Ki‐67 antibody (Abcam, ab16667) overnight and further incubated with a secondary antibody for another 1 h. A DAB peroxidase substrate (Beyotime) was added for signal visualization. Finally, slices were counterstained with hematoxylin (Sigma‐Aldrich) before observation under a fluorescent microscope.

### 2.10. TUNEL Staining

The slices were baked in a 60°C oven for 30 min, deparaffinized in xylene, and rehydrated through a graded ethanol series. Proteinase K was added and incubated with the slices for 30 min. After washing, the slices were stained with TUNEL solution (Roche Diagnostics, Indianapolis, US) at 37°C for 1 h and followed by staining with DAB at room temperature for 10 min. Afterward, cells were photographed under a fluorescent microscope.

### 2.11. Data Analysis

Data from at least three independent experiments were presented as mean ± standard deviation (SD). GraphPad Prism 8.0 (Inc., Chicago, USA) was applied for result analysis. Differences between two groups were assessed using an unpaired two‐tailed Student′s *t*‐test. For comparisons among more than two groups, one‐way analysis of variance (ANOVA) was applied, followed by Tukey’s post hoc test for multiple comparisons. When the *p* value was less than 0.05, the data were considered as statistically significant.

## 3. Results

### 3.1. DLGAP5 was Abnormally Overexpressed in LUAD Cell Lines

**FIGURE 1:**
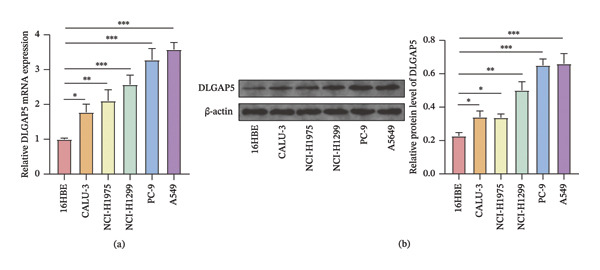
DLGAP5 was abnormally overexpressed in LUAD cell lines (a‐b) RT‐qPCR and western blot analysis for the quantification of the mRNA transcript and protein levels of DLGAP5 in 16HBE cells and LUAD cell lines including CALU‐3, PC‐9, NCI‐H1975, A549, and NCI‐H1299 cells. ^∗^ *p* < 0.05, ^∗∗^ *p* < 0.01, ^∗∗∗^ *p* < 0.001.

Previous evidence has shown that DLGAP5 was significantly highly expressed in various malignant cancer types and plays an oncogene role [10, 21, 22]. In this investigation, we first verified the transcript of DLGAP5 in LUAD cells. As discovered in Figure 1a–1b, compared to normal pulmonary epithelial cells (16HBE), the mRNA and protein level of DLGAP5 was memorably highly expressed in CALU‐3, PC‐9, NCI‐H1975, A549, and NCI‐H1299 cells. Among them, PC‐9 and A549 cells showed higher DLGAP1 levels than other LUAD cells. Thus, PC‐9 and A549 cells were chosen to subsequent experiments.

### 3.2. Repression of DLGAP5 Inhibited the Proliferation and Facilitated Apoptosis of LUAD Cells

To unveil the regulatory roles of DLGAP5 in LUAD cells, the shRNA against DLGAP5 (sh‐DLGAP5) was transfected to knock down the DLGAP5 molecule. Compared to the sh‐NC group, sh‐DLGAP5 transfection observably decreased DLGAP5 mRNA and protein expression (Figure 2a–2b). Then, both CCK‐8 and EdU assays demonstrated that the proliferative capability of LUAD cells was remarkably suppressed after DLGAP5 downregulation (Figure 2c–2d). Moreover, flow cytometry detection also displayed that silencing of DLGAP5 greatly elevated the apoptotic rate of PC‐9 and A549 cells compared to the sh‐NC group (Figure 2e). These outcomes confirmed that loss of DLGAP5 exerted an antitumor role in vitro.

FIGURE 2Loss of DLGAP5 inhibited proliferation and induced apoptosis in vitro. shRNA against DLGAP5 (sh‐DLGAP5) and the negative control (sh‐NC) were transfected into PC‐9 and A549 cells, respectively. (a‐b) RT‐qPCR and western blot analysis validated the silencing efficiency of DLGAP5. (c‐d) CCK‐8 and EdU staining assessed the proliferation of PC‐9 and A549 cells. (e) Flow cytometry evaluated the apoptosis of PC‐9 and A549 cells. ^∗∗^ *p* < 0.01, ^∗∗∗^ *p* < 0.001.

**Figure:**
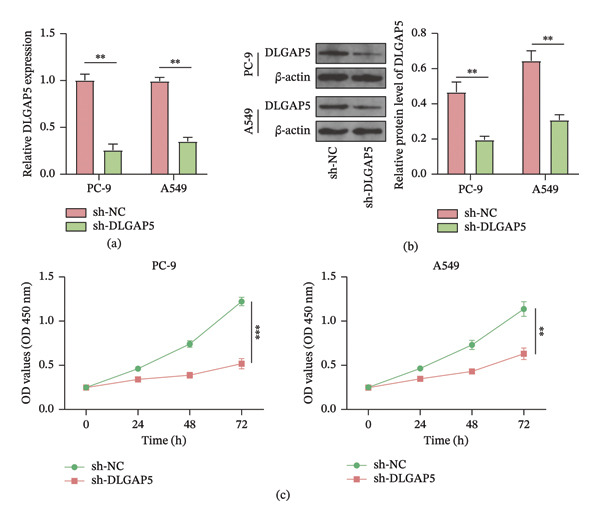

**Figure:**
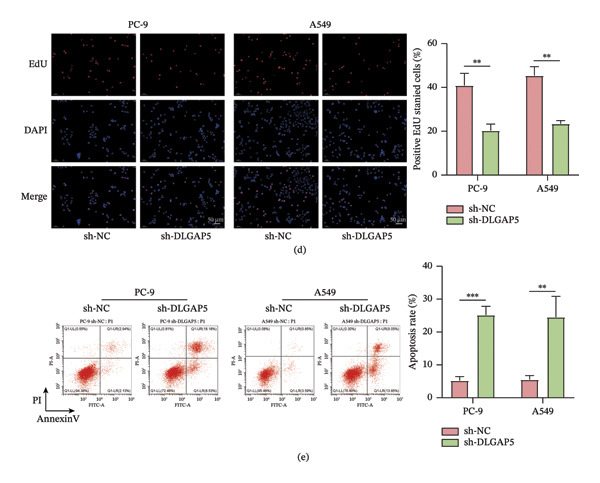

### 3.3. DLGAP5 Knockdown Downregulated the Autophagy Activity in LUAD Cells

**FIGURE 3:**
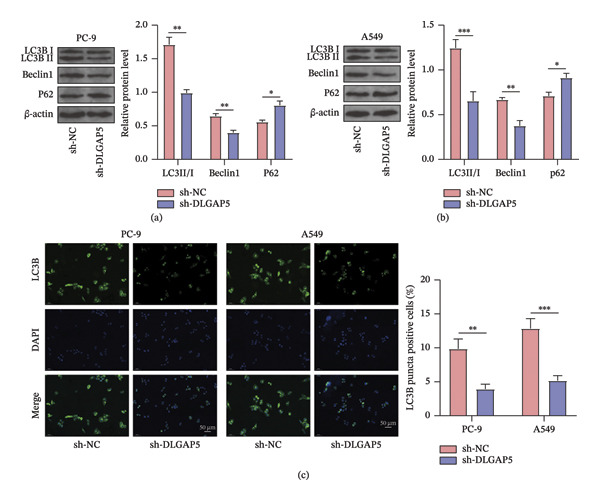
DLGAP5 knockdown downregulated the autophagy activity in LUAD cells. sh‐DLGAP5 and sh‐NC were transfected into PC‐9 and A549 cells, respectively. (a‐b) Western blot was applied to examine LC3II/I, Beclin1, and p62 protein levels. (c) Immunofluorescence assay verified LC3B expression. ^∗^ *p* < 0.05, ^∗∗^ *p* < 0.01, ^∗∗∗^ *p* < 0.001.

Autophagy plays an indispensable role in controlling the survival of tumor cells [23]. Here, we aimed to investigate whether DLGAP5 was involved in the regulation of autophagy activity in LUAD cells. As uncovered in Figure 3a–3b, it was observed that knockdown of DLGAP5 reduced the LC3II/I ratio and Beclin‐1 protein levels but elevated the p62 protein level in LUAD cells. Moreover, immunofluorescence staining also showed that knockdown of DLGAP5 strikingly weakened the fluorescence intensity of LC3B (Figure 3c). Thus, knockdown of DLGAP5 repressed autophagy activity in LUAD cells.

### 3.4. DLGAP5 Regulated the Proliferation and Apoptosis of LUAD Cells by Activating Autophagy Activity

This part was designed to explore the impacts of autophagy on DLGAP1‐mediated LUAD cell proliferation and apoptosis. The overexpressing plasmids of DLGAP5 (OV‐DLGAP5) were transfected to establish DLGAP5‐overexpressing cells. Results presented that compared to the OV‐NC group, OV‐DLGAP5 plasmid transfection greatly enhanced DLGAP5 mRNA and protein levels, and 3‐MA treatment had no significant effect on DLGAP5 expression (Figure 4a–4b). In addition, compared to the OV‐NC group, DLGAP5 upregulation remarkably enhanced the LC3II/I ratio and Beclin1 level but declined the p62 level in PC‐9 and A549 cells, whereas these alterations were mostly reversed by 3‐MA treatment (Figure 4c). Subsequently, enforced expression of DLGAP5 led to a significant increase in cell viability and decrease in cell apoptosis, whereas these effects were greatly mitigated by 3‐MA administration (Figure 4d–4e). These data demonstrated that autophagic flux contributes to DLGAP5‐mediated malignant phenotypes in LUAD.

FIGURE 4DLGAP5 regulated the proliferation and apoptosis of LUAD cells by activating autophagy activity. PC‐9 and A549 cells were transfected with OV‐NC or OV‐DLGAP5 with/without 3‐MA cotreatment. (a‐b) RT‐qPCR and western blot assays quantified DLGAP5 mRNA and protein level. (c) Western blot tested LC3II/I, Beclin1, and p62 protein levels. (d) CCK‐8 was conducted to examine cell viability. (e) Flow cytometry assay was performed to analyze cell apoptosis. ^∗^ *p* < 0.05, ^∗∗^ *p* < 0.01, ^∗∗∗^ *p* < 0.001.

**Figure:**
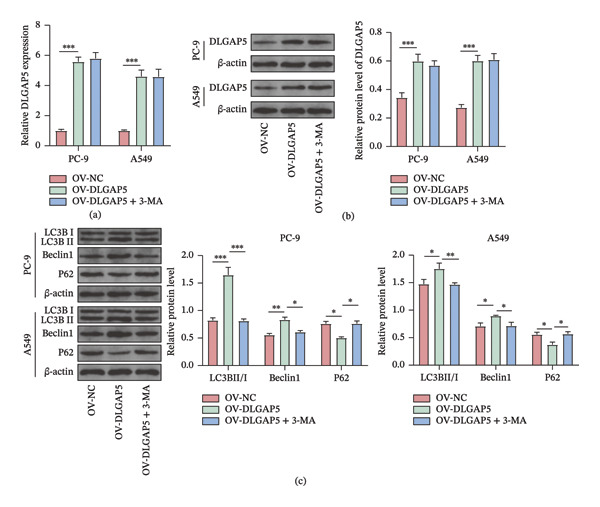

**Figure:**
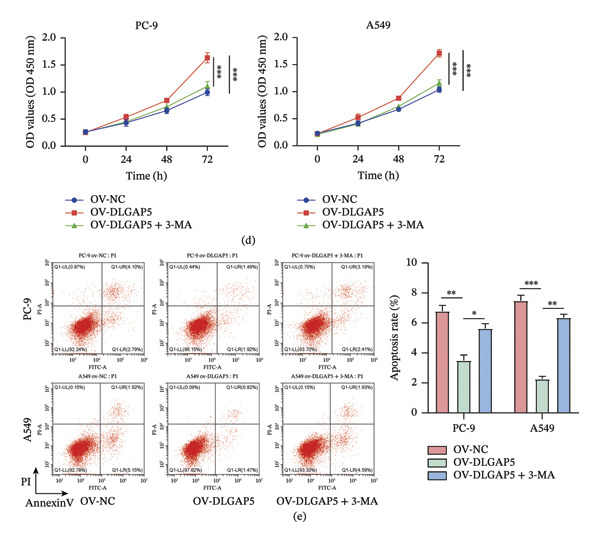

### 3.5. Downregulation of DLGAP5 Inhibited Tumor Growth in Vivo

**FIGURE 5:**
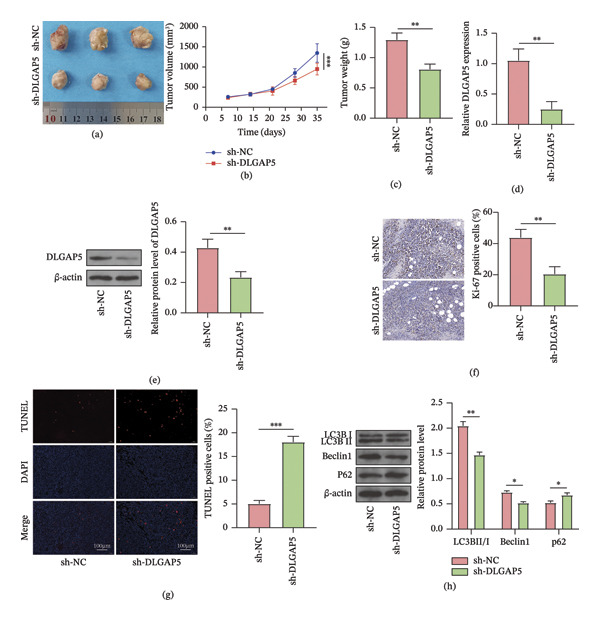
Downregulation of DLGAP5 inhibited tumor growth in vivo A549 cells expressing sh‐NC or sh‐DLGAP5 were injected into the flank of male BALB/c nude mice (*n* = 5 per group). Postinjection of 5 weeks, mice were sacrificed for harvesting the subcutaneous tumors. (a) Representative images of tumors from each group mice. (b) The tumor volume change in each group of mice. (c) Tumor weight of tumor from each group of mice. (d‐e) RT‐qPCR and western blot determined the mRNA and protein levels of DLGAP5. (f) Representative images of Ki‐67 the immunohistochemical test. (g) Representative images of TUNEL staining. (h) Western blot calculated the protein levels of LC3II/I, Beclin1, and p62. ^∗^ *p* < 0.05, ^∗∗^ *p* < 0.01, ^∗∗∗^ *p* < 0.001.

To further validate the in vitro findings, we established a tumor xenograft model. Compared to the sh‐NC group, the tumor weight and tumor volume were dramatically repressed after DLGAP5 inhibition (Figure 5a–5c). Meanwhile, DLGAP5 was markedly decreased in tumor tissues of the sh‐DLGAP5 group than that in the sh‐NC group (Figure 5d–5e). Immunohistochemical results demonstrated that DLGAP5 silencing significantly reduced the expression of Ki‐67 in tumor tissues (Figure 5f). The TUNEL‐labeled apoptotic cells in the sh‐DLGAP5 group were greatly enhanced than those in the sh‐NC group (Figure 5g). Moreover, autophagy‐related proteins were detected by western blot assay. Results described that DLGAP5 inhibition resulted in a decrease of LC3II/I and Beclin1 and an increase of p62, compared to the sh‐NC group (Figure 5h). These data disclosed that DLGAP5 downregulation suppressed tumor growth and autophagy activity in LUAD.

## 4. Discussion

Cancer is one of the most common and threatening diseases worldwide and is regarded as the “second biggest killer” of humans [24]. Clinically, LUAD has the highest incidence in lung cancer, and its clinical outcomes remain not satisfactory, which is largely due to tumor heterogeneity, late diagnosis, and high aggressiveness. Therefore, deciphering the specific mechanism of LUAD is priority to finding new diagnostic and therapeutic targets. In the present work, we examined the function of DLGAP5 in autophagy regulation and malignant cell phenotypes of LUAD. Our findings highlighted that DLGAP5 was significantly upregulated in LUAD cell lines and played a critical role in proliferation, apoptosis, and autophagy activity in LUAD, suggesting that DLGAP5 might be one of the pathogenic factors in LUAD progression.

As a member of the DLGAP family, DLGAP5 is a Ran GTP–dependent microtubule‐associated protein that participates in the modulation of spindle assembly, chromosomal congression, alignment, segregation, and kinetochore fiber stabilization during mitosis [25]. Its expression varies regularly throughout the cell cycle, peaking in the G2/M phase [26]. Given these critical roles in cell division, DLGAP5 has been implicated in various oncogenic processes, including metastasis, apoptosis, immune infiltration, and chemotherapy resistance [11, 25, 27]. Consistent with its proposed oncogenic function, in vitro experiments showed that loss of DLGAP5 reduced the capabilities of proliferation, migration, and invasion in endometrial cancer cells [25], as well as enhanced chemosensitivity in breast cancer [28] and acute myeloid leukemia [11]. In LUAD, DLGAP5 expression was higher, and its depletion led to the decrease of cell growth, cell cycle, and metastasis [14]. In line with and extending this context, our present work demonstrated that DLGAP5 was greatly upregulated in LUAD cell lines, and silencing of DLGAP5 could repress the proliferation and apoptosis inhibition of tumor cells in vitro and in vivo. Furthermore, DLGAP5 also exhibited prognostic value in multiple human tumors (e.g., endometrial cancer [9], colorectal cancer [10], gastric cancer [8], breast cancer [22], and lung cancer [29]), and its upregulation was associated with poorer survival in LUAD [14, 29]. Clinical validation of DLGAP5 expression and its correlation with prognosis in our own LUAD cohort remains a focus of future work. Additionally, the specific molecular mechanisms by which DLGAP5 promotes LUAD progression remain incompletely understood, and this needs to further exploration.

Our results demonstrated that DLGAP5 exerted its oncogenic function in LUAD by activating autophagy activity, which in turn inhibited LUAD cell survival. This finding was supported by the evidence that DLGAP5 knockdown led to the decrease of the autophagy‐related LC3‐II/I ratio and Beclin1 expression while increasing p62 expression. The opposite effects were found in DLGAP5‐overexpressing LUAD cells. More importantly, 3‐MA, an autophagy inhibitor, remarkably abolished the promoting roles of DLGAP5 overexpression on proliferation and apoptosis inhibition. These findings supported that DLGAP5 acted as the upstream of the autophagic cascade to regulate the carcinogenic effect on LUAD cells. As examined in previous reports, autophagy and apoptosis are two interrelated processes, and autophagy could prevent the apoptotic response of tumor cells caused by anticancer drugs or targeted therapies [30, 31]. Similar findings were also proved in LUAD cells, revealing that the autophagy activation protected LUAD cells from apoptosis [32] and cytotoxic effects of gemcitabine [1]. Overall, our findings specified that targeting the DLGAP5‐induced autophagy subpopulation might be a more precise therapeutic strategy.

## 5. Conclusion

**FIGURE 6:**
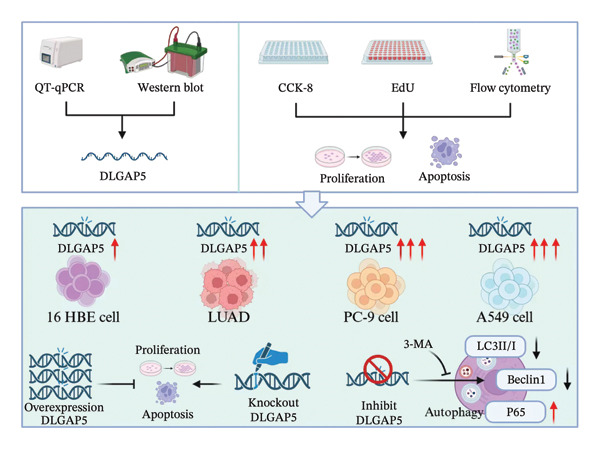
DLGAP5 drives lung adenocarcinoma cell growth by elevating autophagy activity DLGAP5 could contribute to proliferation and impede apoptosis of LUAD cells by activating autophagy, which elucidated that targeting DLGAP5 might be a promising approach for LUAD treatment.

Overall, the present study elucidated a novel signaling pathway of DLGAP5 promoting the occurrence and development of LUAD and described that DLGAP5 promoted autophagy activity to enhance the prosurvival pathway of LUAD cells (Figure 6). These findings indicated that inhibition of DLGAP5 represents a promising approach for LUAD treatment.

## Funding

This study was supported by the Hunan Provincial Health Commission Project (202203022853) and Hunan Provincial Clinical medical technology innovation guide project (2021SK50807).

## Ethics Statement

This study was approved by the Ethics Committee of the Second People’s Hospital of Hunan Province/Brain Hospital of Hunan Province (No. 2021K025).

## Conflicts of Interest

The authors declare no conflicts of interest.

## Data Availability

Data are available on request from the authors.

## References

[1] Fu L. , Li Z. , Wu Y. et al., Hsa-miR-195-5p Inhibits Autophagy and Gemcitabine Resistance of Lung Adenocarcinoma Cells via E2F7/CEP55, Biochemical Genetics. (2023) 61, no. 4, 1528–1547, 10.1007/s10528-023-10330-y. 36658310

[2] Liang W. , Zhang L. , Jiang G. et al., Development and Validation of a Nomogram for Predicting Survival in Patients with Resected non-small-cell Lung Cancer, Journal of Clinical Oncology. (2015) 33, no. 8, 861–869, 10.1200/JCO.2014.56.6661. 25624438

[3] Thai A. A. , Solomon B. J. , Sequist L. V. , Gainor J. F. , and Heist R. S. , Lung Cancer, Lancet. (2021) 398, no. 10299, 535–554, 10.1016/S0140-6736(21)00312-3. 34273294

[4] Ettinger D. S. , Wood D. E. , Aggarwal C. et al., NCCN Guidelines Insights: Non-Small Cell Lung Cancer, Version 1.2020, Journal of the National Comprehensive Cancer Network. (2019) 17, no. 12, 1464–1472, 10.6004/jnccn.2019.0059. 31805526

[5] Siegel R. L. , Miller K. D. , and Jemal A. , Cancer Statistics, 2019, CA: A Cancer Journal for Clinicians. (2019) 69, no. 1, 7–34, 10.3322/caac.21551. 30620402

[6] Hirsch F. R. , Scagliotti G. V. , Mulshine J. L. et al., Lung Cancer: Current Therapies and New Targeted Treatments, Lancet. (2017) 389, no. 10066, 299–311, 10.1016/S0140-6736(16)30958-8. 27574741

[7] Zhang Y. , Tan L. , Yang Q. , Li C. , and Liou Y. C. , The microtubule-associated Protein HURP Recruits the Centrosomal Protein TACC3 to Regulate K-fiber Formation and Support Chromosome Congression, Journal of Biological Chemistry. (2018) 293, no. 40, 15733–15747, 10.1074/jbc.RA118.003676. 30054275 PMC6177585

[8] Li K. , Fu X. , Wu P. , Zhaxi B. , Luo H. , and Li Q. , DLG7/DLGAP5 as a Potential Therapeutic Target in Gastric Cancer, Chinese Medical Journal. (2022) 135, no. 13, 1616–1618, 10.1097/CM9.0000000000001859. 35075051 PMC9532032

[9] Zheng R. , Shi Z. , Li W. , Yu J. , Wang Y. , and Zhou Q. , Identification and Prognostic Value of DLGAP5 in Endometrial Cancer, PeerJ. (2020) 8, 10.7717/peerj.10433. PMC7703392 33312770

[10] Branchi V. , García S. A. , Radhakrishnan P. et al., Prognostic Value of DLGAP5 in Colorectal Cancer, International Journal of Colorectal Disease. (2019) 34, no. 8, 1455–1465, 10.1007/s00384-019-03339-6. 31286215

[11] Shi M. , Guo H. , Bai Y. et al., Upregulated mitosis-associated Genes CENPE, CENPF, and DLGAP5 Predict Poor Prognosis and Chemotherapy Resistance of Acute Myeloid Leukemia, Cancer Biomarkers. (2022) 35, no. 1, 11–25, 10.3233/CBM-203170. 35634845 PMC12364220

[12] Hatfield K. J. , Reikvam H. , and Bruserud O. , Identification of a Subset of Patients with Acute Myeloid Leukemia Characterized by long-term in Vitro Proliferation and Altered Cell Cycle Regulation of the Leukemic Cells, Expert Opinion on Therapeutic Targets. (2014) 18, no. 11, 1237–1251, 10.1517/14728222.2014.957671. 25200484

[13] Zhang H. , Liu Y. , Tang S. et al., Knockdown of DLGAP5 Suppresses Cell Proliferation, Induces G(2)/M Phase Arrest and Apoptosis in Ovarian Cancer, Experimental and Therapeutic Medicine. (2021) 22, no. 5, 10.3892/etm.2021.10680. PMC8438692 34539841

[14] Tang X. , Zhou H. , and Liu Y. , High Expression of DLGAP5 Indicates Poor Prognosis and Immunotherapy in Lung Adenocarcinoma and Promotes Proliferation Through Regulation of the Cell Cycle, Disease Markers. (2023) 2023, 9292536–20, 10.1155/2023/9292536. 36712920 PMC9879687

[15] Singharajkomron N. , Yodsurang V. , Seephan S. et al., Evaluating the Expression and Prognostic Value of Genes Encoding Microtubule-Associated Proteins in Lung Cancer, International Journal of Molecular Sciences. (2022) 23, 10.3390/ijms232314724. PMC9738182 36499051

[16] Onorati A. V. , Dyczynski M. , Ojha R. , and Amaravadi R. K. , Targeting Autophagy in Cancer, Cancer. (2018) 124, no. 16, 3307–3318, 10.1002/cncr.31335. 29671878 PMC6108917

[17] Klionsky D. J. , Petroni G. , Amaravadi R. K. et al., Autophagy in Major Human Diseases, EMBO Journal. (2021) 40, no. 19, 10.15252/embj.2021108863. PMC8488577 34459017

[18] Gao J. , Lu F. , Yan J. et al., The Role of radiotherapy-related Autophagy Genes in the Prognosis and Immune Infiltration in Lung Adenocarcinoma, Frontiers in Immunology. (2022) 13, 10.3389/fimmu.2022.992626. PMC9606704 36311724

[19] Zhang K. , Chen J. , Li C. et al., Exosome-Mediated Transfer of SNHG7 Enhances Docetaxel Resistance in Lung Adenocarcinoma, Cancer Letters. (2022) 526, 142–154, 10.1016/j.canlet.2021.10.029. 34715254

[20] Xu W. , Zhang M. , Li Y. et al., YAP Manipulates Proliferation via PTEN/AKT/mTOR-mediated Autophagy in Lung Adenocarcinomas, Cancer Cell International. (2021) 21, no. 1, 10.1186/s12935-020-01688-9. PMC7791871 33413409

[21] Tang N. , Dou X. , You X. , Shi Q. , Ke M. , and Liu G. , Pan-Cancer Analysis of the Oncogenic Role of Discs Large Homolog Associated Protein 5 (DLGAP5) in Human Tumors, Cancer Cell International. (2021) 21, no. 1, 10.1186/s12935-021-02155-9. PMC8399833 34454476

[22] Xu T. , Dong M. , Li H. , Zhang R. , and Li X. , Elevated Mrna Expression Levels of DLGAP5 are Associated with Poor Prognosis in Breast Cancer, Oncology Letters. (2020) 19, 4053–4065, 10.3892/ol.2020.11533. 32391106 PMC7204629

[23] Poillet-Perez L. and White E. , Role of Tumor and Host Autophagy in Cancer Metabolism, Genes & Development. (2019) 33, no. 12, 610–619, 10.1101/gad.325514.119. 31160394 PMC6546058

[24] Newman C. E. , Gray R. , Brener L. et al., One Size Fits All? the Discursive Framing of Cultural Difference in Health Professional Accounts of Providing Cancer Care to Aboriginal People, Ethnicity and Health. (2013) 18, no. 4, 433–447, 10.1080/13557858.2012.754408. 23297651

[25] Chen R. , Liu J. , Hu J. , Li C. , Liu Y. , and Pan W. , DLGAP5 Knockdown Inactivates the wnt/Beta-Catenin Signal to Repress Endometrial Cancer Cell Malignant Activities, Environmental Toxicology. (2023) 38, no. 3, 685–693, 10.1002/tox.23720. 36454672

[26] Hsu J. M. , Lee Y. C. , Yu C. T. , and Huang C. Y. , Fbx7 Functions in the SCF Complex Regulating Cdk1-cyclin B-phosphorylated Hepatoma up-regulated Protein (HURP) Proteolysis by a proline-rich Region, Journal of Biological Chemistry. (2004) 279, no. 31, 32592–32602, 10.1074/jbc.M404950200. 15145941

[27] Huang R. , Liu J. , Li H. et al., Identification of Hub Genes and Their Correlation with Immune Infiltration Cells in Hepatocellular Carcinoma Based on GEO and TCGA Databases, Frontiers in Genetics. (2021) 12, 10.3389/fgene.2021.647353. PMC8120231 33995482

[28] Zhang X. , Pan Y. , Fu H. , and Zhang J. , Nucleolar and Spindle Associated Protein 1 (NUSAP1) Inhibits Cell Proliferation and Enhances Susceptibility to Epirubicin in Invasive Breast Cancer Cells by Regulating Cyclin D Kinase (CDK1) and DLGAP5 Expression, Medical Science Monitor. (2018) 24, 8553–8564, 10.12659/MSM.910364. 30476929 PMC6278864

[29] Shi Y. X. , Yin J. Y. , Shen Y. , Zhang W. , Zhou H. H. , and Liu Z. Q. , Genome-Scale Analysis Identifies NEK2, DLGAP5 and ECT2 as Promising Diagnostic and Prognostic Biomarkers in Human Lung Cancer, Scientific Reports. (2017) 7, no. 1, 10.1038/s41598-017-08615-5. PMC5556079 28808310

[30] Mulcahy Levy J. M. and Thorburn A. , Autophagy in Cancer: Moving from Understanding Mechanism to Improving Therapy Responses in Patients, Cell Death and Differentiation. (2020) 27, no. 3, 843–857, 10.1038/s41418-019-0474-7. 31836831 PMC7206017

[31] D′Arcy M. S. , Cell Death: a Review of the Major Forms of Apoptosis, Necrosis and Autophagy, Cell Biology International. (2019) 43, no. 6, 582–592, 10.1002/cbin.11137. 30958602

[32] Zhang Y. , Yao E. , Liu Y. et al., FUT2 Facilitates Autophagy and Suppresses Apoptosis via p53 and JNK Signaling in Lung Adenocarcinoma Cells, Cells. (2022) 11, no. 24, 10.3390/cells11244031. PMC9776918 36552800

